# Effect of Recycling on the Thermal and Rheological Properties of PP/MWCNT Composites Used as Liner Materials

**DOI:** 10.3390/polym17162178

**Published:** 2025-08-08

**Authors:** Attila Bata, Ferenc Ronkay, Caizhi Zhang, Péter Gerse

**Affiliations:** 1 Department of Innovative Vehicles and Materials, GAMF Faculty of Engineering and Computer Science, John von Neumann University, Izsáki út 10., H-6000 Kecskemét, Hungary; bata.attila@nje.hu; 2 Jászberény Campus, Eszterházy Károly Catholic University, Rákóczi út 53., H-5100 Jászberény, Hungary; ronkay.ferenc@uni-eszterhazy.hu; 3 The State Key Laboratory of Mechanical Transmissions for Advanced Equipment, College of Mechanical and Vehicle Engineering, Chongqing Automotive Collaborative Innovation Centre, Chongqing University, Chongqing 400044, China; czzhang@cqu.edu.cn

**Keywords:** recycling, polypropylene, carbon nanotubes, nanocomposites, type IV liner material, hydrogen storage

## Abstract

In this study, we developed polypropylene-based nanocomposites using different (0.3, 0.5, and 1 wt%) fillers of multi-walled carbon nanotubes (MWCNTs), with a particular focus on their applicability as lining materials for Type IV hydrogen storage tanks. The aim of this research was to improve the thermal stability and rheological behavior of PP, while also evaluating the recyclability of the resulting composites in order to support sustainability goals. A realistic recycling approach was simulated by producing original and regranulated (REG) samples using a twin-screw extruder. Thermal analysis showed that the incorporation of MWCNTs promoted crystallization, increasing both the degree of crystallinity and lamellar thickness, which are beneficial factors in terms of reducing gas permeability. Rheological tests showed increased storage and loss moduli in both nanocomposites and their recycled counterparts, especially at low frequencies. It is noteworthy that in REG samples with 0.3 and 1 wt% content, the zero-shear viscosity increased by approximately 50% and 90%, respectively, compared to pure PP. In our research, we produced nanocomposites that could offer significant advances in the field of hydrogen storage and liner materials, while the results of the regranulated composites could further enhance the sustainability of our materials.

## 1. Introduction

Nowadays, hydrogen and hydrogen storage are receiving increased attention as they can contribute to mitigating global warming. Therefore, enormous efforts have been made to develop technologies for the production, transport, and storage of hydrogen, as well as for its conversion into usable energy [1]. The advantage of hydrogen is that it contains the highest mass of energy compared to other chemical fuels and enables high-power, large-scale energy storage [2,3,4]. It is an ideal solution for managing the intermittent nature of renewable energy sources. However, its disadvantage is its low volumetric energy density. Hydrogen storage is a key element in the development and growth of hydrogen and fuel technologies for portable/stationary energy supply and transport [5,6]. Polypropylene (PP) is a widely used polymer due to its excellent price–performance ratio and is becoming increasingly popular in the field of hydrogen storage due to its excellent gas barrier properties, excellent mechanical properties, and thermal stability [7,8].

Type IV composite tanks used in hydrogen-powered vehicles must meet strict requirements. The inner polymer lining of the tank must be thermally and mechanically resistant and have adequate gas barrier properties to prevent premature leakage of hydrogen. The described properties can be significantly improved by using various fillers, in particular MWCNTs [9,10].

Multi-walled carbon nanotubes have excellent gas barrier properties due to the impermeable atomic structure of graphene, its unique two-dimensional geometry, and high aspect ratio [11]. They are ideal as fillers for the production of container lining nanocomposites. Previous reports have confirmed the excellent gas barrier performance of graphene-based polymer nanocomposites against the most common gases, namely N_2_, CO_2_, and O_2_ [12].

**Figure 1:**
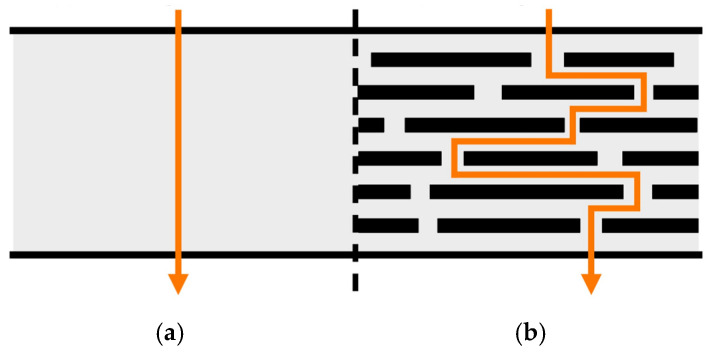
Path of gas molecules through (**a**) unfilled and (**b**) filled polymers.

The primary explanation for the improvement in gas barrier properties is that gas molecules must travel a much more complex path to bypass the filler, thereby reducing the permeability of the material, as shown in Figure 1 [13,14].

At the same time, numerous studies have shown that the increased crystallinity of polymers resulting from the addition of nanomaterials plays a significant role in reducing the gas permeability of nanocomposites [15,16,17,18].

However, to date, there is no literature available on the effect of nanomaterials on the rheological properties and how these influence the permeability of the material. However, numerous studies agree that the interfacial interaction between the filler and the polymer matrix and the proper dispersion of the filler are crucial for improving mechanical and thermal properties and reducing permeability [14,19].

Studies investigating the rheological properties of MWCNT-filled PP nanocomposites consistently find that, with increasing filler content, MWCNT networks form in the PP matrix and interactions between the nanotubes become dominant, leading to changes in the properties of the composite and improvements in its mechanical properties [20,21,22].

In his research [23], Verma investigated the effect of MWCNTs on viscoelastic parameters such as complex viscosity (η*), storage modulus (G’), and loss modulus (G”). The incorporation of MWCNTs into the polymer matrix resulted in higher complex viscosity, storage modulus, and loss modulus compared to the pure polymer, especially in the low frequency range, which indicates a liquid–solid transition in terms of viscoelastic properties.

Lee et al. [24] also investigated the effect of adding MWCNT and a compatibilizer (PP-g-MA) on the rheological and electrical properties of MWCNT/PP nanocomposites. The shear viscosity and yield stress values increased with increasing MWCNT content in the low shear rate range due to increased interaction between MWCNT particles. However, the rheological and electrical properties of PP-g-MA-modified composites with high MWCNT content did not improve compared to PP/MWCNT nanocomposites. It can be concluded that PP-g-MA does not play an important role in improving the interaction between the PP matrix and MWCNT particles.

Yetgin [25] found in his study that all samples examined exhibited Newtonian behavior in the low shear stress range. At higher stresses, a transition range follows, where Newtonian behavior is replaced by non-Newtonian behavior. This transition range defines the linear viscoelastic (LVE) range of the samples, where the storage modulus value in the Newtonian plane is called the Plato modulus (G’p). He found that the value of G’p increases with the addition of MWCNT. The viscoelastic properties of PP/MWCNT nanocomposites, i.e., the storage and loss moduli, also increased significantly with the addition of MWCNT to the PP matrix, especially in the low frequency range [25].

Gupta et al. [21] also investigated random copolymer MWCNT composites and found that both the storage and loss moduli increased significantly with the addition of MWCNT to the PPCP matrix, especially in the low frequency range [21]. PPCP/MWCNT composites showed similar behavior up to a CNT content of 0.4 V% (the slopes of G’ and G” do not change significantly as a function of the circular frequency). However, at CNT contents above 0.4 V%, this final behavior disappears and the dependence of G’ and G” on the circular frequency weakens. This phenomenon has been referred to as a transition from liquid-like to solid-like behavior [26,27,28,29].

The aim of our research is to investigate the thermal and rheological properties of low-filler PP/MWCNT nanocomposites and their regranulates. The special polypropylene used for the tests is a type of raw material that may be suitable for the production of the lining layer of Type IV hydrogen tanks. In addition, with a focus on sustainability, we investigated the recyclability of these materials.

Based on our previous research [19] and the results found in the literature [30,31], the mechanical, time-dependent mechanical, and dynamic mechanical properties of the composites proved to be favorable. Following these positive experiences, our goal is to examine the material system in more detail from a thermal and rheological point of view, thereby broadening the range of properties relevant to hydrogen storage.

Our long-term goal is to manufacture our own vessels if the material proves to be suitable, and to carry out application-oriented tests such as hydrogen permeability and pressure resistance, which could form the basis for a subsequent publication on its usability in the field of hydrogen storage.

## 2. Materials and Methods

### 2.1. Raw Materials

The matrix polymer was a polypropylene homopolymer (TIPPLEN H880, MOL Petrochemicals Ltd., Budapest, Hungary). It had a high molar mass (Mw: 478,865 g/mol; Pd: 6.35) and a low melt index (MFI (230 °C; 2.16 kg) = 0.25 g/10 min).

Nanocyl SA PLASTICYL PP2001 masterbatch was used as a reinforcing material. According to the technical data sheet, the PP/MWCNT masterbatch contains 20% by weight of carbon nanotubes.

### 2.2. Sample Preparation

The nanocomposites and regranulates were produced on a twin-screw extruder (type: LTE26-44, Thailand). The equipment has a twin-screw housing, where the length of each roller module is 4D, i.e., 104 mm. The L/D ratio is 44. The screw diameter is 26 mm. The temperature zones were set to 180–210 °C and the speed to 200 rpm according to the manufacturer’s recommendations.

To produce the PP/MWCNT REG composites, the starting materials were regranulated at a higher speed of 400 rpm. Our aim was to subject the regranulated mixtures to the highest possible shear stress so that they would be exposed to conditions similar to those encountered during actual recycling.

#### 2.2.1. Description of Thermal Testing Equipment and Procedures

Differential scanning calorimetry (DSC) analytical equipment (TA Instruments, Q200, New Castle, DE, USA) was used to investigate melting and crystallization behavior. DSC was used to determine the percentage of partially crystalline fractions in unfilled/filled composites and regranulates. The following relationship was used to determine the degree of crystallinity (X_c_):

(1) $$X_{c}=\frac{{∆H}_{m}}{{∆H}_{m}^{0}}\;*\;100\;[\%]$$

where ${∆H}_{m}$ is the melt enthalpy of the sample during the second heating (J/g). ∆H^0^_m_ is the thermodynamic melt enthalpy of 100% crystalline PP (209 J/g) [30]. For all samples, we used the same procedure, heating the sample from 30 °C to 230 °C in a neutral (nitrogen) atmosphere at 5 °C/min. The first heating cycle shows the temperature history of the tested samples and serves as a basis for checking the technology. This is followed by a slow, controlled cooling at a rate of 5 °C/min, which is slow compared to the manufacturing process, during which the material can develop its “most favorable” structure. This is followed by another heating cycle, also at a rate of 5 °C/min, during which the curve shows the values characteristic of the material.

Besides the thermal behavior of PP/MWCNT, the lamella thickness was also calculated. The lamella thickness provides information about the crystalline structure and the homogeneity of the filler [32]. If the distance between the lamellae varies, we can infer a more homogeneous distribution in the matrix material. The hypothesis related to the shape of the melt enthalpy of the DSC curve ΔH = f (T) has been considered by several researchers. Lamellae are structural elements formed during the melt cooling of partially crystalline polymers as the three-dimensional system grows.

Lamellar fibrils are formed by the radial growth of crystal systems, with fibrils branching to a greater or lesser extent. Larger units, such as spherulites, are found in most semi-crystalline polymers. Especially polymers with lower crystal growth rates, such as polypropylene (about 20 μw/win). In general, the thickness of the lamellae is of the order of about 10 nm [32,33].

After the DSC measurements were performed, the lamella thickness was determined using the Thomson–Gibbs relationship:

(2) $$T_{m}=T_{m}^{0}\left(1-\frac{2\sigma_{e}}{l\Delta H_{f}}\right)$$

where $\left[T_{m}\right]$ is the melting temperature of the polymer under investigation, [$T_{m}^{0}]$ is the equilibrium melting temperature, [l] is the thickness of the lamellae, $[\Delta H_{f}$] is the enthalpy of melting of the perfect crystal, and ${[\sigma }_{e\;}]$ is the surface free energy.

The thickness distribution of the crystalline lamellae can be determined from the results of the DSC curves using the following equation:

(3) $$f\left(l\right)=\frac{1}{M}\frac{dM}{dl}$$

where the thickness of the polymer lamellae is related to the melting temperature of the single crystal ${[T}_{m\;}]$ according to the following equation:

(4) $$l=\frac{2\sigma_{e}T_{m}^{0}}{\Delta H_{f}(T_{m}^{0}-T_{m})}$$

and

(5) $$dM=\frac{dE}{dT}\frac{dT}{\Delta H_{f}}p_{c}$$

where $\left[p_{c}\right]$ is the density of the crystalline phase, and [*dE*] is the energy required to melt *dM* of crystalline mass over the temperature range *T* to *T + dT*

It follows from Equations (4) and (5):

(6) $$\frac{dM}{M}=\frac{dE}{dT}\frac{dT}{{\Delta H}_{f}M}p_{c}$$

where the temperature change [dT] is as follows:

(7) $$dT=dl\frac{{\left(T_{m}^{0}-T_{m}\right)}^{2}}{T_{m}^{0}}\frac{{\Delta H}_{f}}{2\sigma_{e}}$$

And the thickness distribution can be expressed as follows:

(8) $$\frac{1}{M}\frac{dM}{dl}=\frac{dE/dT{\left(T_{m}^{0}-T_{m}\right)}^{2}p_{c}}{2\sigma_{e}T_{m}^{0}M}$$

Evaluation of the following equation based on DSC measurements

(9) $$\frac{dE}{dT}\frac{1}{M}\left[\frac{J}{℃}\frac{1}{\mathrm{kg}}\right]=Heat\;Flow\left[\frac{J}{\mathrm{kg}\cdot \min}\right]/V_{cooling/heating}\left[\frac{℃}{\min}\right]$$

The parameters used to describe the crystallization of isotactic polypropylene monoclinic (α-form) for the determination of lamella thickness are given in Table 1 [34].

#### 2.2.2. Description of Equipment and Measurement Procedures Used for Rheological Tests

Among the rheological measurements, the melt flow index (MFI/MVI) was determined in accordance with ISO 1133-1:2022 [35]; method which is used to determine the melt flow index of thermoplastic raw materials and injection-molded parts/samples in terms of weight (MFI) and volume (MVI). The tests were performed using a Ceast MF20 measuring device. The measurements were performed at 230 °C with a load of 2.16 kg for the mixtures. In the evaluation of the results, the MFI value is only presented, since the MFI and MVI values are proportional to each other, and the conversion factor is the density of the material. The MFI values are presented as an average of 4 measurements.

Measurements in the low deformation rate range were performed using a TA ARES G2 oscillating rheometer (TA Instruments, Inc., New Castle, DE, USA) with a 25 mm diameter plate-plate measuring head and a gap width setting of 1 mm. The dynamic tests were performed at three different temperatures (210–230–250 °C) in the angular frequency range of 0.05–300 rad/s and with a deformation setting of 5%, in each case performing three parallel measurements. Our previous research also confirms that the Carreau–Yasuda model provides one of the most accurate fits.

## 3. Results and Discussion

### 3.1. Evaluation of DSC Results for PP/MWCNT and Their Regranulates

**Figure 2:**
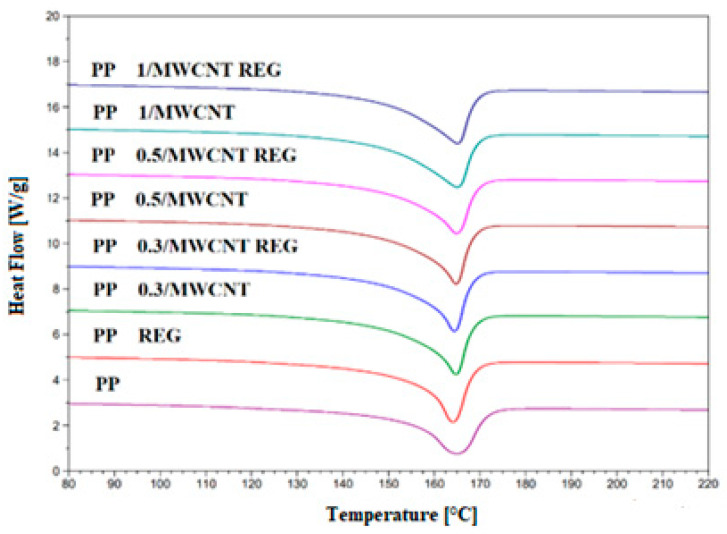
Melting, endothermic curves of PP/MWCNT composites, and their regranulates.

**Figure 3:**
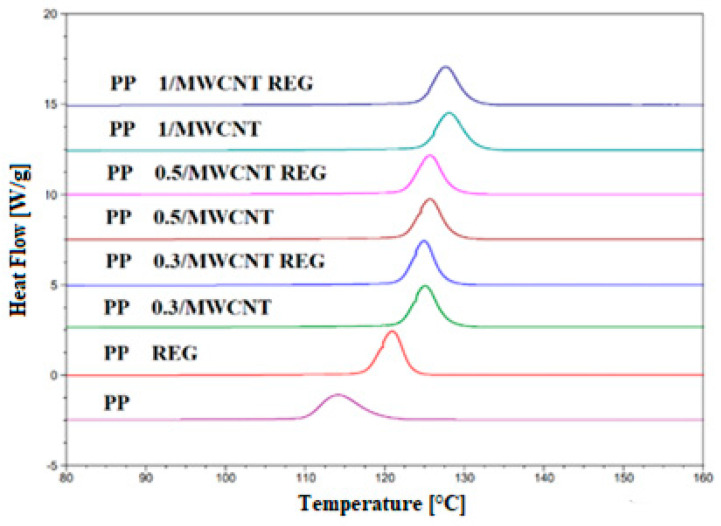
Crystallization, exothermic curves of PP/MWCNT composites, and their regranulates.

Our research has shown that MWCNT facilitates the crystallization of PP, increases the initial crystallization temperature, and increases the adhesion between the MWCNT surface and the polymer chains. The segment mobility is reduced, and MWNCT has a clot-forming effect, as confirmed by crystallinity values (X_c_). DSC curves of the composites and their regranulates are shown in Figure 2 and Figure 3.

In the crystallization curves (Figure 3), the peak temperatures of PP 0.3–0.5/MWCNT and regranulates are nearly identical (~125 °C), while PP 1/MWCNT and regranulates also have a uniform peak temperature 3–4 °C higher. In terms of crystallization onset temperature values, the 1 wt% PP/MWCNT REG shows a difference of ~13 °C compared to the unreinforced PP base material. The crystallization onset and peak temperatures of regranulated PP composites are the same as those of the original PP/MWCNT composites, depending on the MWCNT loading.

Based on the same crystallization peak temperature values, it can be concluded that MWCNT can improve the interphase adhesion between the polymer and nanotube in regranulated composites, which affects the mobility of the segments, and therefore, they have similar crystallization properties as the original PP/MWCNT composites. This shows that MWCNT initiates the crystallization process and that the decrease in molecular weight during reprocessing does not affect the initial and peak crystallization temperatures.

The results of the thermal properties of PP and PP/MWCNT nanocomposites and regranulates are shown in Table 2.

An increase of ~10–15% in the crystallization enthalpy values of the blends was observed compared to the unreinforced PP base material. PP/MWCNT composites and regranulates show an increasing trend in crystalline fraction (X_c_) values compared to the unreinforced PP base material. Regardless of the MWCNT filling ratio, PP/MWCNT and regranulates have on average ~55% crystalline fraction.

**Figure 4:**
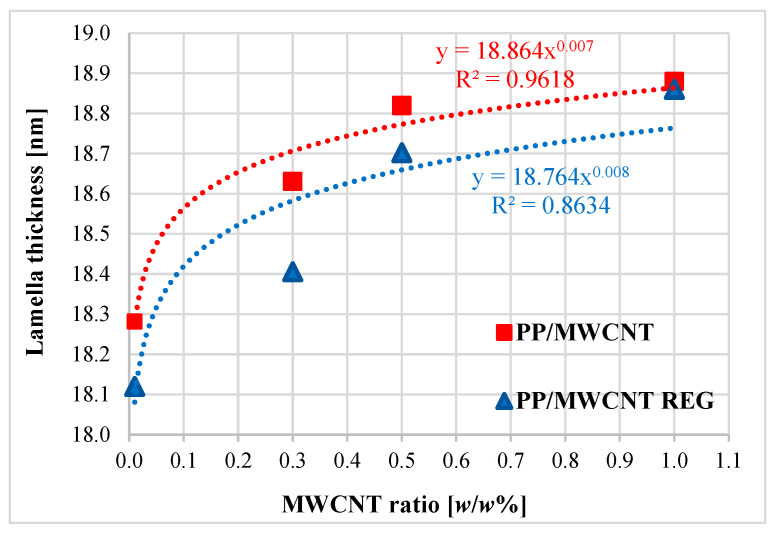
Variation in lamella thickness of PP/MWCNT composites and regranulates in function of MWCNT content.

The theoretical calculation of the thickness distribution of the crystalline lamellae is described in detail in the Materials and Coatings Overview Section, and the results of the calculated lamella thickness are shown in Figure 4.

We found that the folding length of the lamellae increases with the addition of MWCNT. The increasing lamella thickness can be related to the change in crystallization values during the cooling cycle, and the mechanical properties can also be affected by the increasing thickness.

### 3.2. Evaluation of the Rheological Properties of PP/MWCNT and Regranulates

Before presenting the results of the oscillatory rheological tests, we would like to show the melt flow index (MFI) results, which we used to quantify the effect of recycling on the unfilled PP matrix and the composites. Using MFI measurements, we were able to determine the extent of degradation that occurred during processing. If the MFI value increased by at least 25% as a result of recycling, we considered this to be significant degradation, which is approximately equivalent to the extent that can be expected under industrial conditions. The measured MFI value (at 230 °C and 2.16 kg load) was 0.255 g/10 min for unreinforced PP and 0.599 g/10 min for recycled PP, representing an increase of approximately 43%. This confirms that the tested mixtures and their recycled versions realistically represent the degradation that occurs during industrial recycling.

Measurements with an oscillating rheometer over a low shear rate range are suitable for determining zero viscosity. The early part of the viscosity–deformation rate curve was fitted to the polymers using the Carreau–Yasuda model, which describes the viscous flow behavior well over a range of very low to very high shear rates.

**Figure 5:**
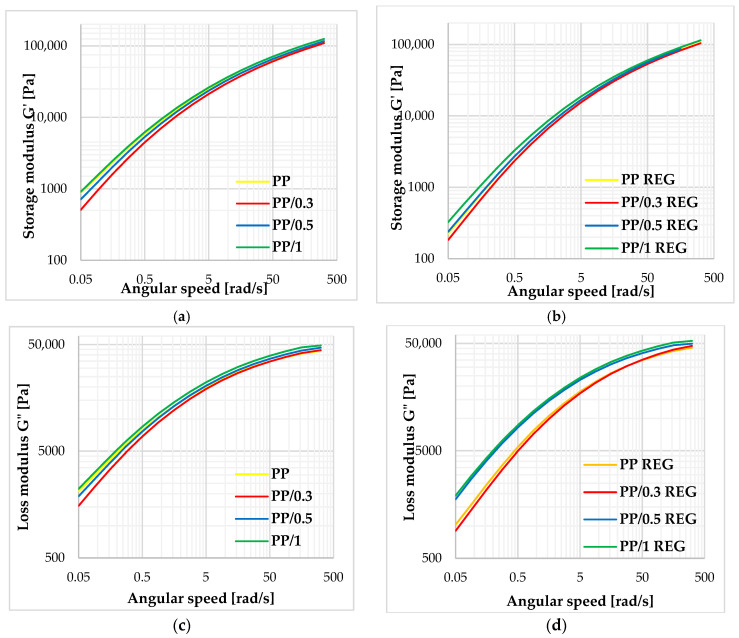
Storage (**a**,**b**) and loss modulus (**c**,**d**) of PP, PP/MWCNT, and regranulates.

Figure 5 shows the evolution of the storage and loss modulus as a function of angular velocity at 230 °C.

The storage and loss modulus of PP/MWCNT composites and regranulates were also increased compared to PP and PP REG materials over the frequency range investigated. The composite regranulates do not reach the modulus values of the unfilled PP material for any of the reinforcements, but the strengthening and stiffening effect is clearly observed compared to the PP REG material.

**Figure 6:**
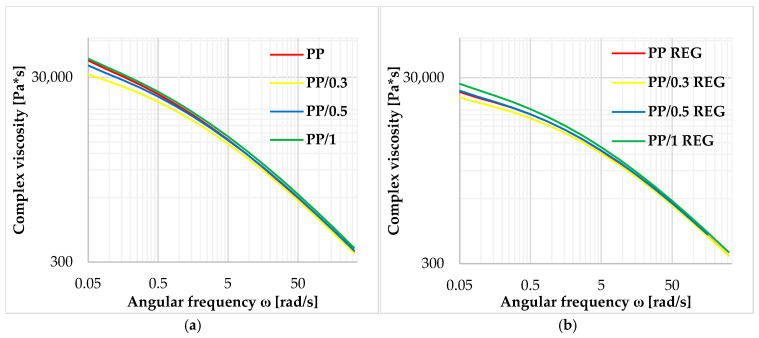
Complex viscosity curves of PP, PP/MWCNT (**a**), and regranulate (**b**) at 230 °C.

Also, the amplification effect can be seen in the complex viscosity values of the tested materials shown in Figure 6.

It is found that the complex viscosity values increase with the addition of MWCNT, indicating that MWCNT increases the melt viscosity in the low deformation rate range. In the high shear rate range, the curves are smoothed, and their viscosities deviate minimally.

**Figure 7:**
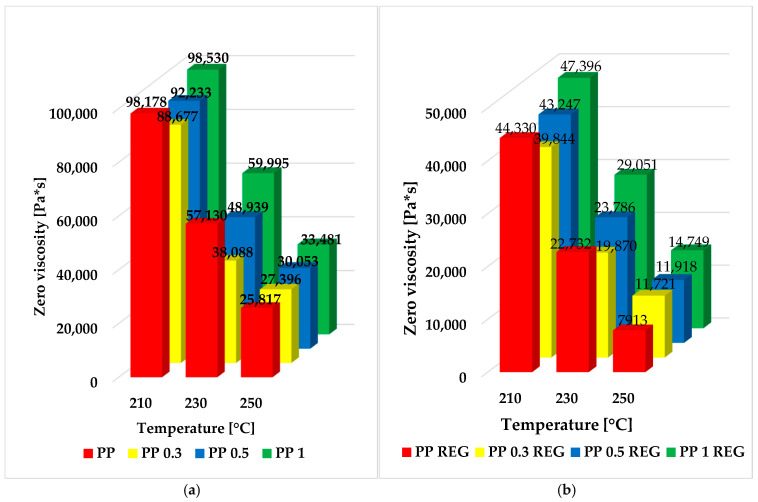
Variation in the zero viscosity values of PP, PP/MWCNT (**a**), and regranulate (**b**) over a wide temperature range (210–230–250 °C).

The zero viscosity values of PP/MWCNT and regranulates were also investigated over a wider temperature range of 210, 230, and 250 °C. These results are shown in detail in Figure 7.

The unfilled PP base material and regranulate have a higher zero viscosity in the lower temperature range (210–230 °C) than composites with 0.3–0.5/MWCNT content. Although the reinforcing effect increases with MWCNT content, only the 1 wt% blend reaches the zero-viscosity value of the unreinforced PP base material in the lower measurement ranges (210–230 °C). However, the results of the measurements at 250 °C show that for composites and regranulates, the loading of 0.3 *w*/*w*% already exceeds the values of PP and PP REG.

At higher temperatures (250 °C), due to the higher mobility of the molecular chains, the MWCNT lattice structure is more inhibited by the segmental parts striving for a new equilibrium state, resulting in a higher zero viscosity value.

**Figure 8:**
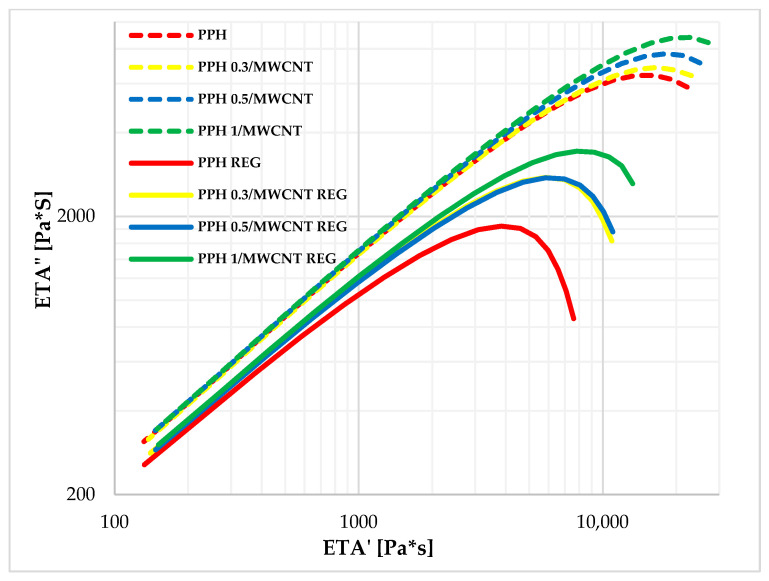
Cole–Cole diagrams of PP, PP/MWCNT, and regranulates at 250 °C.

The Cole–Cole diagrams of PP/MWCNT composites show that they follow the curves for the PP base material and exhibit increasing storage modulus values depending on the MWCNT content. No structural change is observed between the polymer matrix–nanotube, and therefore, it can be stated that up to 1 *w*/*w*% MWCNT loading, aggregation has no effect on the structural properties. Similar behavior is observed for PP/MWCNT REG composites; however, depending on the filler (MWCNT) content, larger variations are observed between regranulated blends. The values of zero viscosity are 0.3–0.5 *w*/*w*%, showing a close correlation with the extrapolated zero viscosity values. The Cole–Cole curves for PP/MWCNT composites and regranulates are illustrated in Figure 8.

It can be observed that the MWCT is homogeneously distributed in both PP and PP REG mixtures. The critical storage modulus value increases continuously with MWCNT charge. It is also observed that the Cole–Cole curves of PP/MWCNT REG composites are spaced further apart upon reprocessing, but they exhibit similar behavior, and their semicircularity is not distorted.

## 4. Conclusions

Based on our previous research and data from the literature, it can be clearly stated that the mechanical and dynamic properties of PP/MWCNT nanocomposites are promising, especially due to the reinforcing effect of the filler. Based on these results, we considered it justified to investigate the thermal and rheological properties of the system in more detail, as these play a key role in the processability, thermal stability, and ultimate applicability of the material, especially in areas such as hydrogen storage.

From the results of the thermal analysis, it can be concluded that the crystalline melting temperature of PP/MWCNT and its regranulates varied between 164 and 165 °C, the crystalline fraction showed an increase of ~9% compared to the unfilled PP feedstock, thus it was found that the filler has a clot-forming effect. This claim is supported by the fact that we observed an increase of 8–10 °C in the initial crystallization temperature and 11–13 °C in the peak temperature compared to the unfilled PP base material, depending on MWCNT reinforcement. The results also show that MWCNT initiates the crystallization process, and the decrease in molecular weight during reprocessing does not affect the initial and peak crystallization temperatures. The lamella thickness shows an increasing trend.

The storage and loss modulus of PP/MWCNT composites and regranulates are also increased compared to PP and PP REG materials, especially in the low frequency range. Based on the results of PP/MWCNT REG composites at 250 °C, the 0.3 and 0.5 *w*/*w*% regranulated composites show ~50% and 1 *w*/*w*% regranulated blends show nearly ~90% higher zero viscosity values compared to the PP REG base material. The Cole–Cole plots and the extrapolated zero viscosity results show a correlation with each other. From the Cole–Cole diagram results, it can be concluded that the critical storage modulus value increases continuously with MWCNT loading. For PP/MWCNT and regranulates, no structural change is observed up to 1 *w*/*w*% MWCNT reinforcement.

The results show that MWCNT filler retains its reinforcing effect in the PP matrix even after recycling. The developed nanocomposites exhibit excellent thermal stability and improved rheological properties, which may make them suitable for hydrogen storage applications. The complex internal structure created by MWCNT and the increased crystallinity also have a positive effect on gas barrier properties and are expected to reduce the gas permeability of the material.

Therefore, our investigations not only confirm the stability and recyclability of the material system, but also that it is worthwhile continuing to work with this composite system. In the future, we aim to manufacture hydrogen tanks and then carry out tests relevant to their end use, such as hydrogen permeability and pressure resistance tests, in order to fully evaluate the practical applicability of the material in the field of hydrogen storage.

## Figures and Tables

**Table 1:** Parameters characterizing the crystallization of α-iPP [34].

Crystal Modulation	$T_{m}^{0}$ [K]	${\Delta H}_{f}$ $\;[J/{cm}^{3}]$	$\sigma_{e}$ $\;[J/{cm}^{2}]$	$p_{c}$ $\;[g/{cm}^{3}]$
α (monoclinic) iPP	464	196	102.9	0.936

where $T_{m}^{0}$ is the equilibrium melting temperature, $\Delta H_{f}$ is the melting enthalpy of the perfect PP crystal, $\sigma_{e\;}$ is the surface free energy, and $p_{c}$ is the density of the crystalline phase.

**Table 2:** Thermal properties results for PP/MWCNT composites and regranulates.

Composites	Second Heating			Cooling		
	$\Delta H_{m}$ (J/g)	$T_{pm}$ (°C)	$X_{c}$ (J/g)	$\Delta H_{c}$ (J/g)	$T_{pc}$ (°C)	$T_{eic}$ (°C)
PP	105	164.2	50.0	100	119.5	114.1
PP REG	108	164.0	51.7	115	123.5	120.9
PP 0.3/MWCNT	115	164.7	54.9	117	127.9	125.1
PP 0.3/MWCNT REG	114	164.4	54.7	117	127.4	124.9
PP 0.5/MWCNT	115	164.7	55.0	113	128.5	125.7
PP 0.5/MWCNT REG	113	164.8	54.3	111	128.5	125.7
PP 1/MWCNT	117	164.0	55.8	118	131.4	128.1
PP 1/MWCNT REG	115	164.1	54.9	115	130.8	127.7

## Data Availability

The original contributions are included in this article. Further inquiries can be requested from the corresponding author.

## Abbreviations

The following abbreviations are used in this manuscript:

PP	propylene
MWCNT	multi-walled carbon nanotubes
REG	regranulates
LVE	linear viscoelastic
